# Amino Acid Substitutions in NS5 Contribute Differentially to Tembusu Virus Attenuation in Ducklings and Cell Cultures

**DOI:** 10.3390/v13050921

**Published:** 2021-05-16

**Authors:** Xue Sun, Mengxu Sun, Lijiao Zhang, Ziding Yu, Jinxin Li, Wanying Xie, Jingliang Su

**Affiliations:** 1 Key Laboratory of Animal Epidemiology and Zoonosis, The Ministry of Agriculture, College of Veterinary Medicine, China Agricultural University, Beijing 100193, China; shellyfree@126.com (X.S.); freedream@cau.edu.cn (M.S.); yuziding@163.com (Z.Y.); nanan1214@163.com (J.L.); Xiewanying19@163.com (W.X.); 2 Institute of Veterinary Medicine, Jiangsu Academy of Agricultural Sciences, Nanjing 210014, China; Zhang62810003@126.com

**Keywords:** Tembusu virus, attenuation, RNA-dependent RNA polymerase, chimeric viruses, viral replication

## Abstract

Tembusu virus (TMUV), a highly infectious pathogenic flavivirus, causes severe egg-drop and encephalitis in domestic waterfowl, while the determinants responsible for viral pathogenicity are largely unknown. In our previous studies, virulent strain JXSP_2-4_ had been completely attenuated by successive passages in BHK-21 cells and the avirulent strain was designated as JXSP-310. Based on the backbone of JXSP_2-4_, a series of chimeric viruses were generated according to the amino acid substitutions in NS5 and their infectivities were also analyzed in cell cultures and ducklings. The results showed that the viral titers of RNA-dependent RNA polymerase (RdRp) domain-swapped cheimeric mutant (JXSP-310^RdRp^) in cells and ducklings were both markedly decreased compared with JXSP_2-4_, indicating that mutations in the RdRp domain affected viral replication. There are R543K and V711A two amino acid substitutions in the RdRp domain. Further site-directed mutagenesis showed that single-point R543K mutant (JXSP-R543K) exhibited similar replication efficacy compared with JXSP_2-4_ in cells, but the viral loads in JXSP-R543K-infected ducklings were significantly lower than that of JXSP_2-4_ and higher than JXSP-310^RdRp^. Surprisingly, the single-point V711A mutation we introduced rapidly reverted. In addition, qRT-PCR and Western blot confirmed that the mutations in the RdRp domain significantly affected the replication of the virus. Taken together, these results show that R543K substitution in the RdRp domain impairs the in vivo growth of TMUV, but sustaining its attenuated infectivity requires the concurrent presence of the V711A mutation.

## 1. Introduction

Tembusu virus (TMUV) was first isolated from *Culex tritaeniorhynchus* mosquitoes in Malaysia in 1955 [1]. TMUV, a member of the *Ntaya* group in the *Flaviviridae* family, has a 10,990-nucleotide-long single-stranded, positive-sense RNA genome. The large, single precursor polyprotein encoded by the viral genome is processed into three structural proteins: the capsid (C), the premembrane/membrane protein (prM), the envelope (E) and seven nonstructural proteins: NS1, NS2A, NS2B, NS3, NS4A, NS4B and NS5 [1,2,3]. TMUV was associated with the explosive epizootics that occurred in commercial duck flocks in China in 2010 and in Southeast Asian countries later on [4,5,6]. The virus is transmitted among susceptible duck flocks by multiple routes (e.g., mosquito bites, infectious aerosol inhalation and eating contaminated feed) and results in severe egg drop and fatal encephalitis [7,8,9]. Because of the widespread duck farms and diversified duck housing systems in China and Southeastern Asia, vaccination is the preferred prophylactic countermeasure against highly contagious TMUV disease. Live-attenuated TMUV vaccines are expected to have several desirable attributes (e.g., stimulation of durable systemic immunity, production efficiency and ease-of-use) when employed in the large and geographically widespread duck population. Nevertheless, the sudden and dramatic emergence of highly pathogenic TMUV in domestic waterfowl highlights the need for better basic understanding of its evolution and host–virus interactions, which are essential prerequisites for effective and safe vaccines.

Several TMUV isolates have been attenuated by serial passage in cell cultures or in chicken embryos under laboratory conditions [10,11,12,13]. Phenotypic and genetic alterations in these isolates have provided us with a wealth of information for analyzing TMUV-associated virulence determinants and pathogenesis. Attempts have been made to investigate the pathogenesis of TMUV and several mutations located in its E protein have been functionally analyzed [8]. However, other genome regions may also contain virulence factors. Published studies showed that single- or multi-point mutations in NS5, carrying methyltransferase (MTase), Linker and RNA-dependent RNA polymerase (RdRp) domain, affected the speed and fidelity of RNA replication (especially RdRp domain) and, thus, altered viral fitness [14,15,16,17,18].

In 2012, our lab successfully isolated a pathogenic TMUV strain JXSP_2-4_ from ducks [1], meanwhile the sequence and pathogenicity of JXSP_2-4_ had also been analyzed [13]. Thereafter, a completely attenuated virus strain JXSP-310 was generated by long-term passage of JXSP_2-4_ in BHK-21 cells [13]. To uncover the underlying mechanisms of TMUV attenuation, the amino acid sequence alignment between JXSP_2-4_ and JXSP-310 showed that there were 23 amino acid substitutions scattered across the whole open reading frame, particularly six substitutions in NS5 and five in E protein. We proved that amino acid changes in the E structural protein were responsible for altering the fitness of the virus in vitro and in vivo [13,19]. Meanwhile, the chimeric derivative of JXSP_2-4_ carrying the NS5 gene from JXSP-310 reduced the virus infectivity in ducklings [13]. To further determine the critical amino acids in NS5 affecting viral pathogenicity, a series of chimeric mutants were constructed and their pathogenicity were also tested. We found that R543K/V711A substitutions in the viral RdRp domain were crucially important for viral infectivity in ducklings. This discovery allows us to further characterize the specific determinants responsible for the pathogenicity of TMUV.

## 2. Materials and Methods

### 2.1. Cells and Viruses

BHK-21 cells and primary duck embryo fibroblasts (DEFs) were grown in Dulbecco’s modified Eagle’s medium (DMEM) containing 10% fetal bovine serum (FBS) and 1% penicillin/streptomycin at 37 °C with 5% CO_2_. Virus infected and non-infected cell monolayer controls were maintained in DMEM supplemented with 2% FBS. The JXSP_2-4_ duck Tembusu virus strain (GenBank accession no. JQ920423.1) and the attenuated JXSP-310 strain (GenBank accession no. MZ031023) were used in this study [13].

### 2.2. Construction of the Chimeric Virus and Site-Directed Mutagenesis

**Figure 1:**
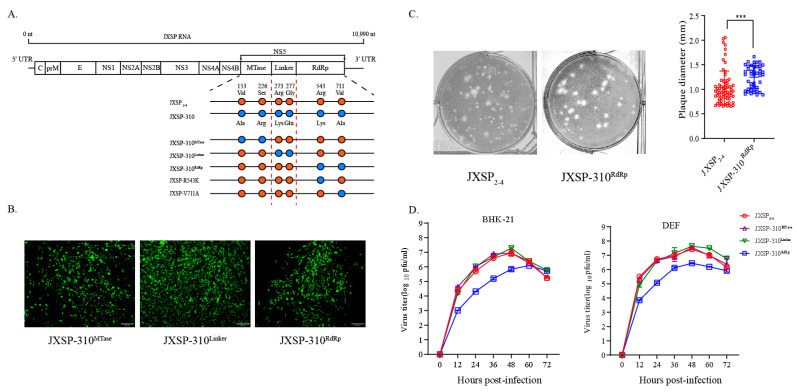
Characteristics of chimeric viruses in vitro. (**A**) Strategy used to construct chimeric and mutant viruses. The JXSP-310^MTase^ chimera contains two mutations (V153 and S226), JXSP-310^Linker^ contains two mutations (R273, G277) and JXSP-310^RdRp^ also contains two mutations (R543, V711). The filled circles represent the parental JXSP_2-4_ (orange) and JXSP-310 (blue) viruses, respectively. (**B**) Immunofluorescence assay. BHK-21 cells were fixed after 48 h infection with the chimeric viruses and stained with an antibody against the E protein. Scale bar 100 μm. (**C**) Plaque phenotypes of JXSP_2-4_ (n = 50) and JXSP-310^RdRp^ (n = 50). The mean ± SD values are shown and the data were tested for statistical significance using the Student’s t test. ***, *p* < 0.001. (**D**) Multi-step growth curves of the chimeric viruses. BHK-21 cells and DEF cells were separately infected with JXSP_2-4_, JXSP-310^MTase^, JXSP-310^Linker^ or JXSP-310^RdRp^, each at an MOI of 0.1. The culture supernatants were collected every 12 h and viral titers were quantified using plaque assay. All experiments were performed in triplicate and the mean ± the SD values are shown.

Chimeric viruses construction and site-directed mutagenesis were conducted as previously described [13]. Briefly, as shown in Figure 1A, the first-strand cDNAs of JXSP_2-4_ and JXSP-310 were used as templates to PCR-amplify five overlapping fragments covering the whole genome with the primers listed in Table 1. Each new modified fragment was cloned into the pEASY Blunt Simple Cloning Vector (TransGen, Beijing, China). The cDNA clones of the three chimeric mutants (JXSP-310^MTase^, JXSP-310^Linker^ and JXSP-310^RdRp^) and two single-point mutants (JXSP-R543K and JXSP-V711A) were produced by fusion PCR. The full-length genomic cDNA was transcribed in vitro using the T7 RiboMAX Express Large Scale RNA Production System (Promega, Fitchburg, WI, USA) according to the manufacturer’s instructions. The transcripts were transfected into 2 × 10^5^ pre-seeded BHK-21 cells with Lipofectamine 3000 Transfection Reagent (Invitrogen, Carlsbad, CA, USA). The supernatants from the RNA-transfected cells were harvested at 72 h post-transfection when typical cytopathic effects were observed. The supernatants were then used to infect fresh BHK-21 cells to amplify the rescued viruses. The sequences of the rescued viruses were confirmed by full-genome sequencing. Viral titers were determined by standard plaque assays in BHK-21 cells. The amplified viral stocks were aliquoted and stored at −80 °C.

### 2.3. Immunofluorescence Assays

BHK-21 cells grown in 12-well plates were infected with the rescued viruses or were mock infected. At 48 h post-infection, the cells were fixed in cold (−20 °C) acetone and methanol (1:1) for 20 min at room temperature. The fixed cells were washed with PBS three times and then incubated with anti-E protein mAb (1:1000 dilution in PBS) at 37 °C for 1 h. After three washes with PBS, the cells were incubated with DyLight488-conjugated goat anti-mouse IgG (Canlifesci Inc., Co., Ltd., Beijing, China) (1:1000 dilution in PBS) at 37 °C for another 1 h. Finally, the plates were washed three times and examined by fluorescence microscopy.

### 2.4. Virus Replication Kinetics

To investigate replication capacity of the chimeric viruses, mutant viruses and their parental strain, BHK-21 cells and DEF cells were grown in 6-well plates to 80% confluence and then infected with each virus at an multiplicity of infection (MOI) of 0.1. After inoculation at 37 °C for 1 h, the supernatants were discarded and the cells were washed three times with PBS. Fresh DMEM (2 mL) and 2% FBS were added. The cell culture supernatants were collected at various time points post-infection. Progeny virus yields were quantitated by plaque assay in BHK-21 cells.

### 2.5. Plaque Assays

Serial 10-fold dilutions of each viral sample were prepared in DMEM (plus 1% FBS) and inoculated onto BHK-21 monolayers in 6-well plates. The plates were incubated at 37 °C for 1 h. After inoculum removal, the cells were washed twice with PBS and overlaid with DMEM containing 1% (wt/vol) low-melting point agarose (Amresco, Houston, TX, USA) and 2% FBS. After further incubation at 37 °C for 72 h, the cells were stained with 0.02% neutral red to visualize the plaques.

### 2.6. Duck Infection Experiments

One-day-old Pekin ducklings, purchased from Beijing Golden Star Duck Co., Ltd., (Beijing, China) were used to examine the virulence of the chimeric and mutant viruses. Before challenge, TMUV negativity was confirmed in the ducklings using a blocking enzyme-linked immunosorbent assay [20]. For the challenge groups, 7-day-old healthy Pekin ducklings were infected subcutaneously with 10^5^ plaque forming units (PFUs) of the parental virus, the chimeric viruses or the mutant viruses. The control group was mock infected with 0.5 mL DMEM. Viral loads were detected in the tissues of five euthanized ducks from each group at various time points. The blood and tissue samples from the heart, liver, spleen, lungs, kidneys, thymus and bursa of Fabricius were collected for viral titer determination. Tissue samples were homogenized and diluted in DMEM to a final concentration of 20% (wt/vol). After three cycles of freeze-thawing, the tissue suspensions were centrifuged (5000× *g* at 4 °C for 20 min). Viral supernatant titers were determined using plaque assay in BHK-21 cells.

### 2.7. RNA Extraction and qRT-PCR Analysis

RNAs in the supernatants from the infected DEF cells and tissue samples were extracted using a SV Total RNA Isolation System (Promega, Fitchburg, WI, USA). cDNAs were synthesized using a reverse transcription system (Promega, Fitchburg, WI, USA) with random primers. Real-time PCR was conducted in a Bio-Rad CFX Connect real-time system using SYBR green SuperReal PreMix Plus (Tiangen, Beijing, China) with primers (forward, 5′-TCATTGATAGAATTTGAGGAG-3′; reverse, 5′-TTCCAATTTGCTTCCAGAGTA-3′) targeting the TMUV E region. A plasmid fragment containing the E region’s genome sequence was used to prepare a standard curve for quantifying viral genome copy numbers. Relative qRT-PCR was performed to detect the transcription level of IFN-α, IFN-β, IFN-γ, IL-6 and IL-1β and the primers used in this study were listed in Table 2.

### 2.8. Western Blot Analysis

DEF cells in 6-well plates, which were separately infected with the parental virus and mutant viruses, were collected at various time points post-infection. Cells were lysed with Cell Lysis Buffer for Western and IP (IP: immunoprecipitation; Beyotime, Shanghai, China). Protein concentrations were determined by the BCA method (Solarbio, Beijing, China). Equal protein amounts were separated by 12% sodium dodecyl sulfate polyacrylamide gel electrophoresis and then transferred from the gel to polyvinylidene difluoride membranes by wet transfer. The membranes were blocked with 5% skimmed milk in PBST (PBS/Tween) at 37 °C for 2 h. The membranes were incubated at 37 °C for 1 h with anti-E protein mAb or anti-β-actin mAb (1:5000 dilution in PBST). After three washes with PBST, the membranes were incubated with secondary antibodies for 1 h at 37 °C. Finally, the protein bands were detected using the BeyoECL Plus Kit after three washes (Beyotime, Shanghai, China).

### 2.9. Statistical Analysis

All data were processed with GraphPad Prism 8 (GraphPad Software Inc., La Jolla, CA, USA). The student’s t-test or two-way multiple ANOVA comparisons test was used to analyze differences between the values from the two groups. A value of *p* < 0.05 was considered statistically significant.

## 3. Results

### 3.1. Replacing the NS5 RdRp Domain Decreases the Growth of the Virus in Cell Cultures

The data from our previous study suggested that amino acid substitutions in NS5 contributed to virulence attenuation of TMUV JXSP_2-4_ [13]. To identify the sub-regions within TMUV NS5 responsible for virulence attenuation, we first constructed three chimeric mutants by individually exchanging the MTase, Linker and RdRp domains in attenuated JXSP-310 with the corresponding sites in the genome of the JXSP_2-4_-derived infectious clone by reverse genetic manipulation. The overall cloning strategy is shown in Figure 1A. The recombinant viruses were detected by immunofluorescence staining with a monoclonal antibody against the TMUV E protein (Figure 1B) and verified by genomic sequencing, resulting in JXSP-310^MTase^, JXSP-310^Linker^ and JXSP-310^RdRp^ chimeric viruses for the MTase, Linker and RdRp domains, respectively. We noted that the JXSP-310^RdRp^ chimeric virus produced larger plaque sizes in BHK-21 cell monolayers than its parental JXSP_2-4_ virus (*p* < 0.001) (Figure 1C), suggesting that the fitness of JXSP-310^RdRp^ might have been altered. We then compared the in vitro replication kinetics of the chimeric viruses with that of parental JXSP_2-4_ using multi-step growth curves. BHK-21 and DEF cells were infected with either chimeric virus or the parental virus at an MOI of 0.1. The JXSP-310^RdRp^ chimeric virus grew significantly slower than the parental virus JXSP_2-4_ in both cell types with 10- to 25-fold lower infectious virus titers and its peak titer lagged by approximately 12 h in BHK-21 cells (Figure 1D). In contrast, JXSP-310^MTase^ and JXSP-310^Linker^ exhibited growth kinetics comparable to those of the parental virus. These results suggested that mutations in the NS5 RdRp domain decreased the replication ability of TMUV in cultured cells. However, the increased plaque size of JXSP-310^RdRp^ was not correlated positively with virus multiplication in cell cultures. 

### 3.2. The In Vivo Infectivity of Chimeric JXSP-310^RdRp^ Is Attenuated

**Figure 2:**
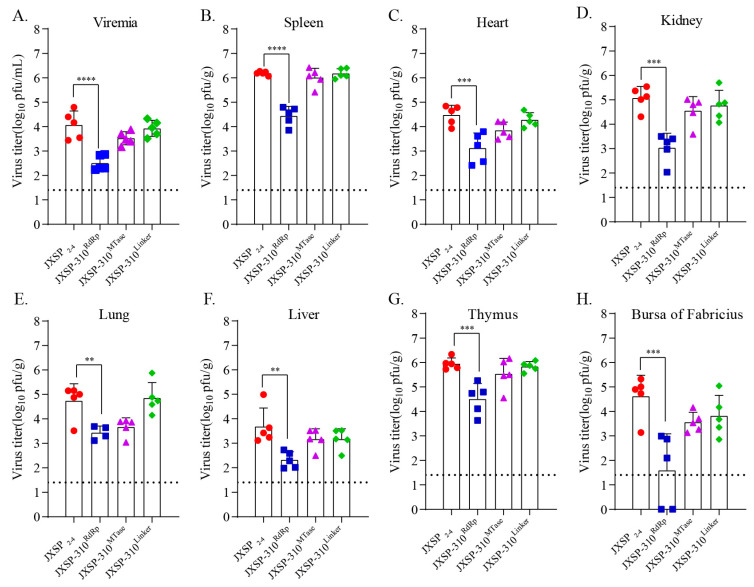
The viral loads of the chimeric viruses at 2 day post-infection. Seven-day-old ducklings were subcutaneously inoculated with JXSP_2-4_, JXSP-310^MTase^, JXSP-310^Linker^ and JXSP-310^RdRp^ (1 × 10^5^ PFU/bird) separately and euthanized 2 days later. Viral loads in the blood (**A**), spleen (**B**), heart (**C**), kidneys (**D**), lungs (**E**), liver (**F**), thymus (**G**) and bursa of Fabricius (**H**) were determined using plaque assay in BHK-21 cells. Data were tested for statistical significance by two-way multiple ANOVA comparisons. Each chimeric virus was compared with JXSP_2-4_ (**, *p* < 0.05; ***, *p* < 0.001; ****, *p* < 0.0001). The dashed line represents the detection limit of the assay.

**Figure 3:**
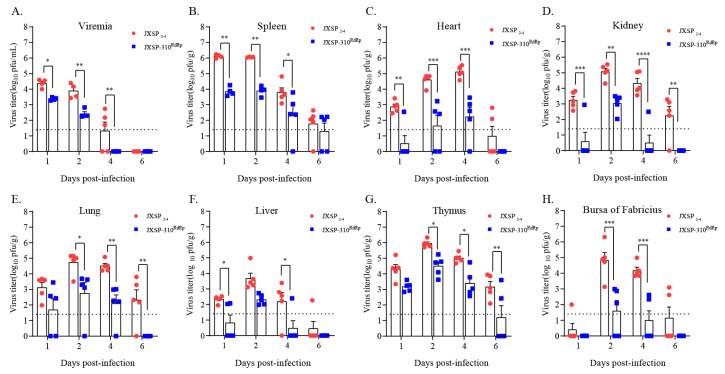
The viral loads of JXSP_2-4_ and JXSP-310^RdRp^ at various time points. Seven-day-old ducklings were inoculated with JXSP_2-4_ and JXSP-310^RdRp^ (1×10^5^ PFU/bird) and then euthanized at day 1, 2, 4 and 6 post-infection. Viral loads in the blood (**A**), spleen (**B**), heart (**C**), kidneys (**D**), lungs (**E**), liver (**F**), thymus (**G**) and bursa of Fabricius (**H**) were determined using plaque assay in BHK-21 cells. The data were tested for statistical significance by two-way multiple ANOVA comparisons (*, *p* < 0.05; **, *p* < 0.01; ***, *p* < 0.001; ****, *p* < 0.0001). The dashed line represents the detection limit of the assay.

To examine the effects of mutations in the aforementioned domains on viral virulence, we initially compared the in vivo infectivity of the chimeric viruses by measuring viral loads in the tissues of 7-day-old ducklings after subcutaneous infection with 10^5^ PFU of virus per duckling (n = 5 per group). Plaque assay quantification showed that the infectious virus loads of JXSP-310^RdRp^ in representative tissues from the inoculated ducklings were 30- to 100-fold lower than those of the parental virus on day 2 post-infection (pi) (Figure 2). No significant difference was observed between the JXSP-310^MTase^- and JXSP-310^Linker^-infected ducklings. To further monitor the in vivo growth kinetics of the JXSP-310^RdRp^ chimeric virus, a group of 7-day-old ducklings (n = 20 per group) were subcutaneously infected with 10^5^ PFU of JXSP-310^RdRp^ and another group of 20 with the JXSP_2-4_ parental virus. Ducklings were sacrificed on days 1, 2, 4 and 6 pi and the viral loads in representative tissues were measured by plaque assays. The infectivity titers in the tested tissues of the JXSP-310^RdRp^-infected ducklings were all significantly lower than those of JXSP_2-4_ (Figure 3). On day 1 pi the viremia and viral titers in the spleen peaked and these values in JXSP-310^RdRp^-infected ducklings were 10-fold and 150-fold lower, respectively, than in the JXSP_2-4_-infected ducklings. On day 2 pi, the viral titers in the other organs peaked and JXSP-310^RdRp^ was significantly attenuated (*p* < 0.01). By day 4 pi, the viral titers in the heart tissues from the JXSP-310^RdRp^-infected ducklings were 200-fold lower than in the JXSP_2-4_-infected ducklings (*p* < 0.001). These results indicate that replacing the RdRp domain in TMUV attenuates its in vivo infectivity.

### 3.3. The NS5 Val711 to Ala Mutation Reverts Rapidly when the R543K Substitution Is Absent

**Figure 4:**
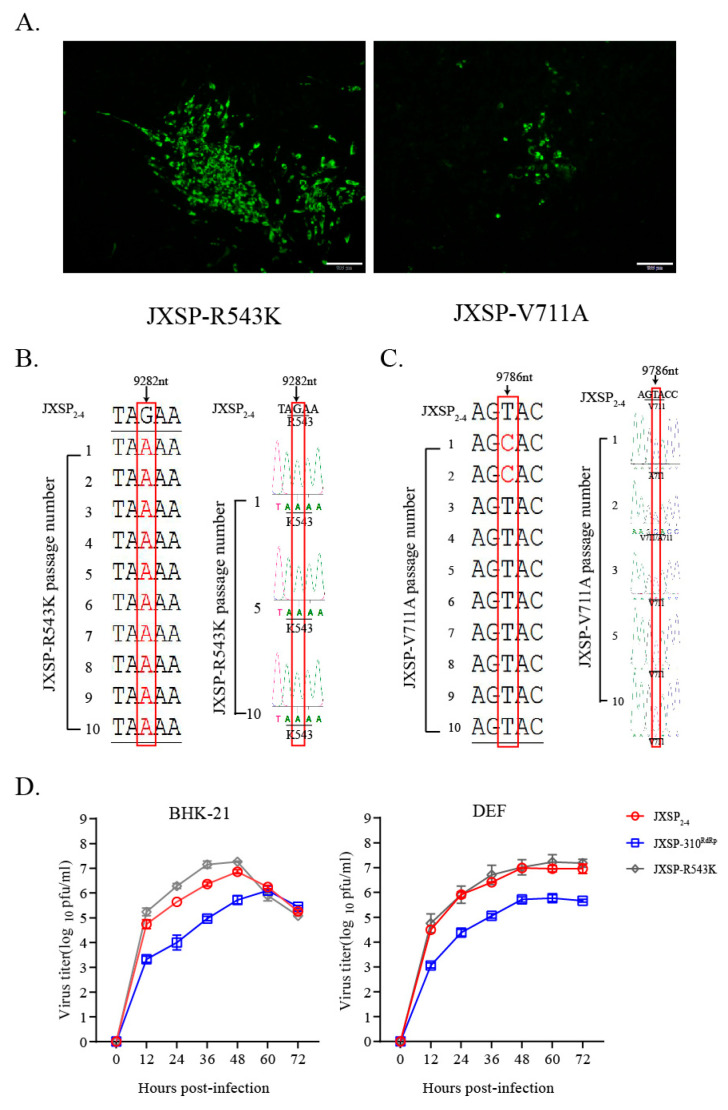
Characterization of mutant viruses in vitro. (**A**) Immunofluorescence assay. BHK-21 cells were fixed at 48 h after infection with either of the rescue viruses and stained with antibody against the E protein. Scale bar 100 μm. (**B**,**C**) Whole genome sequence analysis of 10 serially passaged mutant viruses. The JXSP-R543K mutant virus was stable during serial passage, but the 711-site mutant reverted to Val (GTA). (**D**) Multi-step growth curves of the mutant viruses. BHK-21 cells and DEF cells were infected with JXSP_2-4,_ JXSP-310^RdRp^ and JXSP-R543K (MOI of 0.1). The culture supernatants were collected every 12 h and the viral titers were quantified using plaque assay. All experiments were performed in triplicate and the mean ± SD values are shown.

Because the chimeric JXSP-310^RdRp^ virus consistently exhibited attenuated phenotypes, we determined whether specific amino acid substitutions were associated with these phenotypic alterations. A single arginine to lysine (R543K) or valine to alanine (V711A) in NS5 was introduced into the JXSP_2-4_ genome by site-directed mutagenesis (Figure 4A). Sequence analysis indicated that the R543K mutation site appeared to be genetically stable after 10 serial passages in BHK-21 cells (Figure 4B). When the multi-step growth curves were compared with those from the parental virus, the single-point R543K mutant we constructed exhibited growth kinetics comparable to that of the parental virus in BHK-21 or DEF cells (Figure 4D). This indicated that the R543K substitution alone did not influence viral growth in vitro. However, the Val to Ala mutation (GTA to GCA) reverted to Val (GTA) via a single nucleotide substitution in the V711A mutant after one passage in BHK-21 cells (Figure 4C). Additional attempts at virus rescue resulted in the same reversion. 

### 3.4. The NS5 R543K Mutant Is Less Attenuated In Vivo when the V711A Mutation Is Absent

**Figure 5:**
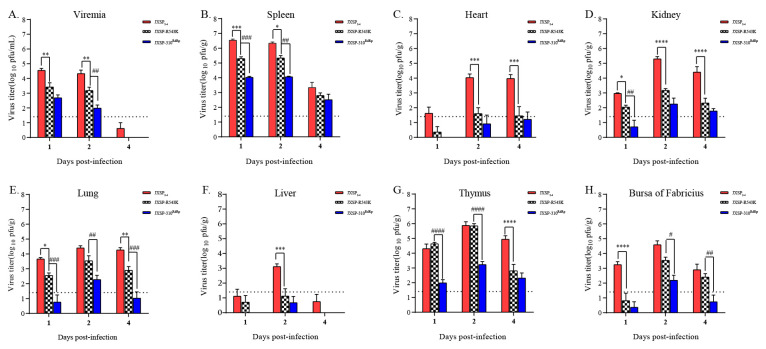
The viral loads of JXSP-R543K, JXSP-310^RdRp^ and JXSP_2-4_ at various time points. Seven-day-old ducklings were inoculated with JXSP_2-4_, JXSP-310^RdRp^ and JXSP-R543K (1 × 10^5^ PFU/bird) separately and euthanized at days 1, 2 and 4 post-infection. Viral titers in the blood (**A**), spleen (**B**), heart (**C**), kidneys (**D**), lungs (**E**), liver (**F**), thymus (**G**) and bursa of Fabricius (**H**) were determined using plaque assay in BHK-21 cells. Asterisks indicate significant differences between JXSP_2-4_ and JXSP-R543K, (*, *p* < 0.05; **, *p* < 0.01; ***, *p* < 0.001; ****, *p* < 0.0001). Number signs indicate significant differences between JXSP-310^RdRp^ and JXSP-R543K (#, *p* < 0.05; ##, *p* < 0.01; ###, *p* < 0.001; ####, *p* < 0.0001). The data were tested for statistical significance by two-way multiple ANOVA comparisons. The dashed line represents the detection limit of the assay.

To investigate whether the single R543K change in NS5 would affect the ability of the virus to replicate in vivo, individual groups of 7-day-old ducklings (n = 15) were subcutaneously infected with JXSP_2-4_, JXSP-310^RdRp^, or JXSP-R543K and the infectivity titers in the representative tissues were quantified by plaque assays. Ducklings infected with JXSP-310^RdRp^ had significantly lower infectious virus titers in their sera and in various organs than those infected with parental JXSP_2-4_ (Figure 5), which were consistent with the abovementioned results. By comparison, the single-point R543K mutant appeared to have a less attenuated replication capacity when compared with the parental virus and JXSP-310^RdRp^. However, the peak viral titers in the serum, spleen, heart and kidneys were significantly lower, although the differences in the lung and thymus were not statistically significant when compared with the parental virus. This suggests that the R543K and V711A mutations in combination confer better in vivo infectivity attenuation. 

**Figure 6:**
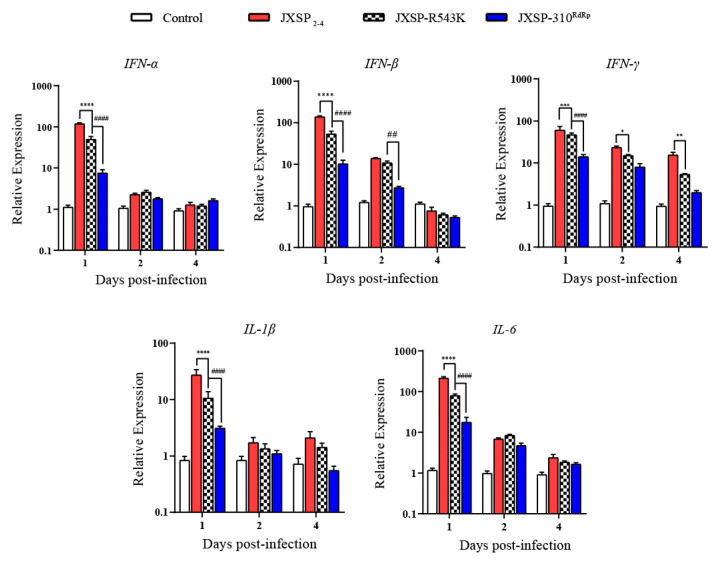
Immune-related gene expression among JXSP_2-4_-, JXSP-310^RdRp^-and JXSP-R543K-infected ducklings. The mRNA levels of IFN-α, IFN-β, IFN-γ, IL-1β and IL-6 in the spleens were analyzed by qRT-PCR and normalized to GADPH. Asterisks indicate significant differences between JXSP_2-4_ and JXSP-R543K, (*, *p* < 0.05; **, *p* < 0.01; ***, *p* < 0.001; ****, *p* < 0.0001). Number signs indicate significant differences between JXSP-310^RdRp^ and JXSP-R543K (##, *p* < 0.01; ####, *p* < 0.0001). The data were tested for statistical significance by two-way multiple ANOVA comparisons.

To compare the innate immune responses to these viruses, we examined the expression of interferons and early pro-inflammatory cytokines in the spleen by qRT-PCR. As shown in Figure 6, the innate immune responses in ducklings were strongly elicited by infection with TMUV JXSP_2-4_ and the mutant viruses. The increase of transcript abundance in the spleen was positively correlated with the replication levels of these viruses. The magnitude of spleen-tissue interferon (IFN)-α, IFN-β, IFN-γ, interleukin (IL)-1β and IL-6 expression induced by infection with the R543K mutant was significantly lower than those of JXSP_2-4_, but higher than those of the JXSP-310^RdRp^-infected ducklings. Overall, the early induction of interferons and pro-inflammatory cytokines reflected the infectivity profiles of the viruses.

### 3.5. Coupled Mutations of NS5 R543K and V711A Decrease Viral RNA Synthesis

**Figure 7:**
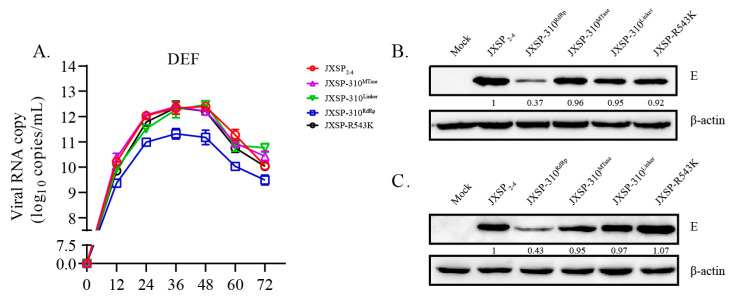
RNA synthesis and protein production of the viruses. (**A**) Quantitative analysis of RNA production in the parental virus, chimeric viruses and the single-site mutant virus by qRT-PCR in DEF cells. (**B**,**C**) Protein lysates from DEF cells collected at 24 hpi and 36 hpi were analyzed by western blotting using antibodies against the E protein and the internal β-actin control. The numbers represent the ratios in comparison with JXSP_2-4_, which were determined by densitometry.

To further analyze the effect of RdRp mutations on virus replication, DEF cells were separately infected with either of the mutated viruses or the parental virus at an MOI of 0.1 and RNA copy numbers were measured by qRT-PCR. The JXSP-310^RdRp^ RNA copy numbers were 5- to 15-fold lower than those of the parental virus (Figure 7A), whereas the RNA copy numbers for chimeric JXSP-310^MTase^ and JXSP-310^Linker^ were similar to those of the parental virus-infected DEF cells, ranging from equal to 2-fold differences. Consistent with the in vitro infectivity observed herein and in common with other chimeric viruses, no significant RNA replication deficiency was observed in the single-point R543K mutant. The RNA copy numbers in the virus-infected DEFs correlated with the infectious virus titers detected by the plaque assays. In agreement with the RNA replication results, the western blot showed that E protein expression level of the JXSP-310^MTase^, JXSP-310^Linker^ and JXSP-R543K was similar to that of JXSP_2-4_ in DEFs at 24 and 36 hpi. However, the E protein production in JXSP-310^RdRp^-infected cells was 2.7-fold and 2.3-fold lower, respectively, than that of JXSP_2-4_ (Figure 7B,C).

## 4. Discussion

Our previous studies showed that 23 amino acid substitutions in multiple genes co-contributed to the full attenuation of this virus [13]. Here, the effects of mutations in NS5 protein on viral attenuation were analyzed. We demonstrated, for the first time, that a single R543K substitution in the RdRp domain of NS5 impaired the infectivity of TMUV in vivo and that the virus was more strongly attenuated when R543K substitution was coupled with the V711A mutation.

NS5, the largest (905 amino acids) and most conserved flavivirus protein, contains three independent domains: N-terminal methyltransferase (MTase) domain, which is involved in RNA capping and prevents host recognition as well as promotes translation [21,22,23]; C-terminal RdRp domain, which is responsible for de novo RNA synthesis [24,25]; and the short Linker in between, which regulates the cross talk between RdRp and MTase dmoains [26]. The critical roles played by NS5 in viral replication and interferon antagonism make it a prime target for antiviral therapy and vaccine development [27,28]. To map the specific amino acid substitutions in the NS5 that contributed to duck TMUV attenuation, we generated a series of chimeric viruses by replacing the individual domains of the wild-type JXSP_2-4_ with those of the attenuated JXSP-310. The in vitro cell cultures and in vivo ducklings infection results showed that replacing JXSP_2-4_ with either the mutated MTase or Linker domain did not significantly alter the chimeric viruses’ phenotypes, whereas the R543K and V711A mutations in the RdRp domain resulted in significant lower infectivity titers when compared with its parental virus. These results demonstrated that mutations in MTase and Linker had no influence on the activity of MTase and the cross-talk between RdRp and MTase. Thus, the adaptive mutations in the RdRp domain selected from laboratory cell culture passages changed the fitness of TMUV.

Generally, the plaque size is determined by virus replication rates and cell-to-cell spread. Previous study showed that PDK53 strain of DENV replicated rapidly, but giving rise to small plaques due to its inability to evade antiviral responses that constrained its spread in primate or rodent cells [29]. Figure 6 showed that JXSP_2-4_-infected ducklings produced higher inflammatory cytokines compared with JXSP-310^RdRp^; thus, the enhanced immune responses might account for the smaller plaque size.

**Figure 8:**
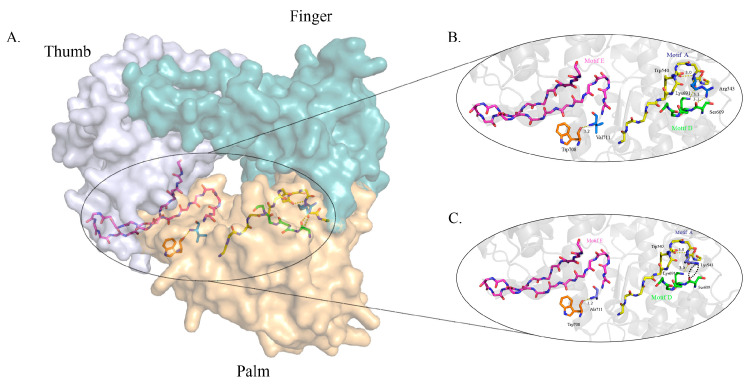
Details of the side-chain interactions between residues R543 and V711 in TMUV RdRp. (**A**) The structure of NS5 RdRp of TMUV shows the fingers, palm and thumb domains colored in blue, grey and orange, respectively. The TMUV NS5 protein model was created online using Swiss-Model with reference to the crystal structure of the JEV NS5 protein (PDB identifier [ID] 4k6m). (**B**) R543 is located in motif A and possibly forms three solid hydrogen bonds with W540, S690 and K691 spatially. V711 is close to motif E and forms one solid hydrogen bond with W708. (**C**) An amino acid mutation at site 543 changes arginine to lysine, which breaks the hydrogen bond between sites 543 and 690. A valine to alanine change at site 711 slightly changes the distance between motif A and residue W708. This figure was generated in PyMol.

Flavivirus RdRp, whose overall shape resembles a right hand with fingers, palm and thumb subdomains, contains seven conserved motifs (A to G) [30,31]. Studies showed that amino acid substitutions in the RdRp domain affected genome replication and disease development [16,32]. In foot-and-mouth disease virus, replacing the conserved residues in the motif A of RdRp resulted in virulence attenuation in mice by altering the polymerase’s fidelity [33]. Predictive structural models of this protein suggest that residue Arg543 is highly conserved within the motif A (YADDTAGWDTR/KIT). The motif A forms the catalytic site in the RdRp with the motif C in the structural palm domain. The single-point R543K mutation in the RdRp described herein did not affect TMUV replication in vitro, as determined by plaque assays and RNA copy quantification. However, significantly lower viremia and viral titers of the mutant were observed in most of the tested parenchymal organs, suggesting that factors other than the physiological condition of the ducklings restricted virus proliferation. One possible explanation is that replacing arginine with lysine partially retains the properties of arginine through conservation of structure; however, the smaller lysine residue may affect interactions with other viral components and possibly host-factor proteins, too. Interchanging lysine and arginine at residue 185 of the PA polymerase of avian H5N1 virus TY165 significantly altered its virulence in mice [34]. Homology modelling analysis predicts that R543 is located in the TMUV RdRp module’s α helix, which forms three hydrogen bonds with residues Trp540, Ser690 and Lys691. Contrastingly, the R543K substitution is predicted to break the hydrogen bond between Arg543 and Ser690, which is the first residue of motif D (Figure 8). Interestingly, the NS5 R543K mutation was also observed in a recent laboratory attenuated TMUV strain [35].

The second mutated residue Val711 is just before motif E in the thumb subdomain of RdRp. However, this mutation rapidly reverted when the rescued single-point mutant was cultured in BHK-21 cells, implying that the V711A mutation was deleterious to viral proliferation and fitness in cell cultures in the absence of the R543K substitution. We noted that a reverse mutation of alanine to valine in the corresponding site of the duck TMUV Du/CH/LSD/110128 strain was reported to occur during serial passaging in chicken embryos [10]; this Val711 to Ala mutation occurred after 30 passages and the reversion was identified in the 50th-passage virus. This also suggested that there was a strong preference for alanine at this position. This amino acid substitution, however, was stable in the R543K/V711A double mutant. In our previous study, the R543K mutation in JXSP_2-4_ was identified after the 150th passage during BHK-21 cell passaging. The mutation occurred far earlier than the V711A change, which was detected from the 280th passage [13]. The stability of the R543K/V711A double mutant suggests that the R543K mutation somewhat counterbalances the negative effect. It is not clear whether this residue change affects the conformation and/or connection with the palm subdomain because the correct conformation of motif E is important for de novo initiation and elongation efficiency [36]. Although further biochemical studies are needed, we observed that the double mutations caused significantly lower RNA replication and infectivity reduction in infected DEFs. Moreover, the more dramatic attenuation of the R543K/V711A double mutant suggests that the two mutations act in concert to significantly alter the biological properties of TMUV, possibly through the additive effects of these mutations on the functionality of the viral proteins and/or genes. Interestingly, the amiloride-induced coxsackie virus B3 variant with an Ala to Val change at position 372 in the RdRp (3Dpol-A372V), which corresponds to residue 711 in TMUV RdRp, was found to increase enzyme fidelity [37]. In the study by Harrison et al., the A372V mutants of coxsackie B3 virus that were screened by amiloride derivative treatment contained the aspartic acid to glycine substitution in residue 48 of the 2A protein (2A-D48G) and double mutations were found to play a synergistic effect in replication, resulting in higher viral titers in the presence of antiviral compounds [38].

In combination with the phenotypic differences observed between the single-point R543K mutant and the fact that the R543K/V711A double mutant consistently displayed reduced viral replication in vitro and in vivo, it appears that both mutations significantly alter the biological properties of the virus. Identifying the amino acids responsible for virulence attenuation could lead to new insights into viral pathogenesis and help in designing new vaccines for disease control. The virulence attenuation in the RdRp mutants also provides several excellent models with which to gain further knowledge of the molecular properties of viruses of medical and veterinary importance.

## Figures and Tables

**Table 1:** Primers used in the construction of chimeric viruses and single-point mutants.

Primer Name	Sequence (5′-3′)	Application
T7 + 1F	CCCGGGTAATACGACTCACTATAGGGAGAAGTTCATCTGTGTGAACTTATTCC	PCR amplification fragment-1
2459R	GTCGATTGAGCACCCCGTGTC	
2459F	GACACGGGGTGCTCAATCGAC	PCR amplification fragment-2
3514R	TTCCATGCCACCCCCTTGAAA	
3514F	TTTCAAGGGGGTGGCATGGAA	PCR amplification fragment-3
5851R	CGACTATCTATGACCCGTTGC	
5285F	ATAGCGGAAGCACTGAAAGGA	PCR amplification fragment-4
8164R	CAACGCCCCTAGCTAACCATT	
8164F	AATGGTTAGCTAGGGGCGTTG	PCR amplification fragment-5
10990R	AGACTCTGTGTTCTACCACCACCAG CCACACTTTCGGCGATCTGTGCCAA	
7656R	GTTCTGCCAGTTCCCCCTC	MTase domain of NS5 gene substitution
7656F	GAGGGGGAACTGGCAGAAC	
8487R	CTTCACCCTATCAGCGACCA	Linker domain of NS5 gene substitution
8487F	TGGTCGCTGATAGGGTGAAG	
10350R	CAAGACACCTTCACTCCAGC	RdRp domain of NS5 gene substitution
10350F	GCTGGAGTGAAGGTGTCTTG	
9282R1	CTTGGTTATTTTAGTGTCCCA	Mutation PCR of R543(R-K)
9282F2	TGGGACACTAAAATAACCAAG	
9786R1	GCAAAAGGGTGCTTCTTGCCA	Mutation PCR of V711(V-A)
9786F2	TGGCAAGAAGCACCCTTTTGC	

**Table 2:** Primers used in the quantification of duck immune-related gene by qRT-PCR.

Primer Name	Sequence (5′-3′)	Application
IFNα-F	TGTGGTTCTGGAGGAAGTGTTG	IFN-α analysis
IFNα-R	AACCAGCTTCAGCACCACATC	
IFNβ-F	AGGATGTTGAAGAGGTGTTG	IFN-β analysis
IFNβ-R	CTTTTGGACACCGACAAC	
IFNγ-F	AATGACATAGACAAACTGAAAGCTG	IFN-γ analysis
IFNγ-R	CAGGGTAACAATCTGGCTCAG	
IL6-F	TTGAGTCGCTGTGCTATAG	IL-6 analysis
IL6-R	CTCTATCCAGGTCTTATCCG	
IL1β-F	TCGACATCAACCAGAAGTGC	IL-1β analysis
IL1β-R	GAGCTTGTAGCCCTTGATGC	
GADPH-F	AAATTGTCAGCAATGCCTCTTG	GAPDH analysis
GADPH-R	TGGCATGGACAGTGGTCATAA	

## Data Availability

The data presented in this study are available on request from the corresponding author.
